# Photorespiration Enhances Acidification of the Thylakoid Lumen, Reduces the Plastoquinone Pool, and Contributes to the Oxidation of P700 at a Lower Partial Pressure of CO_2_ in Wheat Leaves

**DOI:** 10.3390/plants9030319

**Published:** 2020-03-03

**Authors:** Shinya Wada, Yuji Suzuki, Chikahiro Miyake

**Affiliations:** 1 Department of Biological and Environmental Science, Faculty of Agriculture, Graduate School of Agricultural Science, Kobe University, 1-1 Rokkodai, Nada, Kobe 657-8501, Japan; swada@penguin.kobe-u.ac.jp; 2 Core Research for Environmental Science and Technology, Japan Science and Technology Agency, K’s Goban-Cyo, 7 Goban-Cyo, Chiyoda-Ku, Tokyo 102-0076, Japan; ysuzuki@iwate-u.ac.jp; 3 Faculty of Agriculture, Iwate University, 3-18-8 Ueda, Morioka, Iwate 020-8550, Japan

**Keywords:** photorespiration, photosynthesis, photosystem I, P700 oxidation, reactive oxygen species, reduction-induced suppression of electron flow (RISE)

## Abstract

The oxidation of P700 in photosystem I (PSI) is a robust mechanism that suppresses the production of reactive oxygen species. We researched the contribution of photorespiration to the oxidation of P700 in wheat leaves. We analyzed the effects of changes in partial pressures of CO_2_ and O_2_ on photosynthetic parameters. The electron flux in photosynthetic linear electron flow (LEF) exhibited a positive linear relationship with an origin of zero against the dissipation rate (vH^+^) of electrochromic shift (ECS; ΔpH across thylakoid membrane), indicating that cyclic electron flow around PSI did not contribute to H^+^ usage in photosynthesis/photorespiration. The vH^+^ showed a positive linear relationship with an origin of zero against the H^+^ consumption rates in photosynthesis/photorespiration (JgH^+^). These two linear relationships show that the electron flow in LEF is very efficiently coupled with H^+^ usage in photosynthesis/photorespiration. Lowering the intercellular partial pressure of CO_2_ enhanced the oxidation of P700 with the suppression of LEF. Under photorespiratory conditions, the oxidation of P700 and the reduction of the plastoquinone pool were stimulated with a decrease in JgH^+^, compared to non-photorespiratory conditions. These results indicate that the reduction-induced suppression of electron flow (RISE) suppresses the reduction of oxidized P700 in PSI under photorespiratory conditions. Furthermore, under photorespiratory conditions, ECS was larger and H^+^ conductance was lower against JgH^+^ than those under non-photorespiratory conditions. These results indicate that photorespiration enhances RISE and ΔpH formation by lowering H^+^ conductance, both of which contribute to keeping P700 in a highly oxidized state.

## 1. Introduction

Plants, both wild and cultivated, face the threat of oxidative damage from reactive oxygen species (ROS) when they are exposed to environments in which photosynthesis is suppressed [1]. For example, low temperatures, high temperatures, and dryness promote stomata closure, which reduces photosynthesis abilities [2]. In these circumstances, superoxide radicals (O_2_^−^) can be generated through the photoreduction of O_2_^−^ in photosystem I (PSI), and H_2_O_2_ is generated by the disproportionation of O_2_^−^ [2]. The photoreduction of O_2_ in PSI is regarded as the main ROS-generating process in photosynthetic organisms exposed to environmental stresses [2]. These ROS increase the risk of oxidative damage.

In angiosperms, ROS generation in PSI has been shown to cause oxidative damage [1,3]. To imitate situations in which electrons accumulate on the PSI acceptor side—situations of environmental stress that lowers photosynthesis efficiency and NADP^+^ regeneration efficiency—the leaves of sunflower plants were illuminated intermittently with saturating lights in darkness (repetitive short-pulse (rSP) illumination treatment). This rSP illumination treatment promoted PSI oxidative damage over time. On the other hand, almost no oxidative damage occurred in photosystem II (PSII) [1,3,4]. Under anoxic conditions, the PSI oxidative damage was suppressed [3]. The rSP illumination treatment promoted ROS generation within PSI, which was thought to be the cause of the oxidative damage. Additionally, this PSI damage also lowered the photosynthesis rate [3,5].

The reaction center chlorophyll P700 in PSI drives the photo-oxidation/reduction cycle. Ground state P700 absorbs light and transitions into its excited state (P700*). Then, oxidized P700 (P700^+^) is generated when P700* donates electrons to the electron acceptors in the PSI complex (Ao, A_1_, Fx, and F_A_/F_B_) [6]. When leaves are irradiated with a pulse light, P700^+^ is generated rapidly. However, during the pulse, P700^+^ decreases and P700* accumulates [1,4,7]. P700* accumulation promotes electron transfer from A_0_, A_1_, F_X_, and/or F_A_/F_B_ to O_2_ to produce O_2_^−^. This is the mechanism of ROS generation in PSI by rSP illumination, as well as a molecular mechanism of PSI oxidative damage.

The accumulation of photoexcited P700* implies that the rate-determining step of the P700 photo-oxidation/reduction cycle is the electron transfer reaction from P700* to the electron acceptors on the PSI acceptor side. This has been motivating us to clarify the reason why O_2_-evolving photosynthesis organisms can safely perform photosynthesis under field conditions [1,3,4]. If P700* does not accumulate under pulse light illumination, ROS generation should be suppressed. Therefore, to keep P700* from accumulating, the reduction of P700^+^ in the P700 photo-oxidation/reduction cycle should be the rate-determining step of the cycle. 

In this study, we conducted rSP illumination treatment under steady-state actinic light (AL) conditions [3]. As the intensity of AL increased, the PSI oxidative damage caused by the rSP illumination treatment was lowered. Furthermore, it was found that an increase in AL intensity increased the proportion of P700^+^ in the photo-oxidation reduction cycle of P700 in PSI [3]. We revealed a negative relationship between PSI oxidative damage and P700^+^ accumulation under AL conditions [3]. These results show that P700^+^ accumulation lowers the proportion of P700*, which causes the generation of ROS by pulse illumination.

We clarified that O_2_-evolving photosynthesis organisms suppress ROS generation in PSI through P700 oxidation [1,3,4,7,8,9,10,11,12]. Shimakawa et al. [4], in particular, revealed that a cyanobacterial strain that does not maintain a high level of P700^+^ suffers from rapid PSI oxidative damage under AL illumination. Nearly 30 years ago, it was reported that under conditions with strong light or a low partial pressure of CO_2_ (pCO_2_), i.e., conditions with a reduced photosynthetic efficiency, plants display the oxidation of P700 in PSI [13,14,15,16,17,18,19]. We suggest that P700 oxidation is a robust physiological response for suppressing ROS generation.

Photorespiration is thought to contribute to P700 oxidation [1,20]. For the PSI reaction center chlorophyll P700 to be kept in a higher oxidized state, the regeneration rate of the ground state of P700 in the photo-oxidation/reduction cycle must be limited by the P700^+^ reduction rate. In this study, we attempted to explain the molecular mechanism by which photorespiration facilitates the oxidation of P700. 

## 2. Materials and Methods

### 2.1. Plant Materials and Growth Conditions

The winter wheat cultivar “Norin 61” was used in this study. Seeds were incubated on wet cotton at 4 °C for 3 days to promote synchronized germination. The moistened seeds were grown in a mixture of soil (Metro-Mix 350; Sun Gro Horticulture, Bellevue, WA, USA) and vermiculite (Konan, Osaka, Japan) in pots (7.5 cm length × 7.5 cm width × 6 cm depth). The ratio of soil to vermiculite was 1:1. The plants were grown under standard air-equilibrated conditions in an environmentally controlled chamber set at 25 °C day/20 °C night, with a 16 h light/10 h dark photoperiod and 700–800 µmol photon m^−2^ s^−1^ light intensity. They were watered every other day with 0.1% Hyponex solution (N:P:K = 5:10:5; Hyponex, Osaka, Japan). The plants were grown for at least 6 weeks, and fully expanded, mature leaves were harvested for further analysis.

### 2.2. Gas Exchange, Chlorophyll Fluorescence, P700^+^, Electrochromic Shift, and Spectroscopic Analyses 

Exchanges of CO_2_ and H_2_O were measured using the GFS-3000 system equipped with a 3010-DUAL gas exchange chamber (Walz, Effeltrich, Germany), in which ambient air was saturated with water vapor at 14.0 ± 0.1 °C and the leaf temperature was maintained at 25 ± 2 °C. The photosynthesis rate (A) and dark respiration rate (Rd) were measured. The photosynthesis rate as a function of the intercellular partial pressure of CO_2_ (Ci) was determined. Three plants were used for each experiment. Gas exchange parameters were calculated by the software of the GFS-3000 system, which follows the method of von Caemmerer and Farquhar [21]. 

The chlorophyll fluorescence and P700^+^ in PSI were measured with a DUAL-PAM system (Walz), simultaneously with the gas exchange analysis of GFS-3000 (Walz). The chlorophyll fluorescence parameters were calculated as follows [22]: F_o_, minimum fluorescence from a dark-adapted leaf; F_o_′, minimum fluorescence from a light-adapted leaf; F_m_, maximum fluorescence from a dark-adapted leaf; F_m_′, maximum fluorescence from a light-adapted leaf; Fs, fluorescence emission from a light-adapted leaf; effective quantum yield of PSII, Y(II) = (F_m_′ − Fs)/F_m_′; non-photochemical quenching, non-photochemical quenching (NPQ) = (F_m_ − F_m_’)/F_m_’; and Q_A_ oxidized state (qL) = (F_m_’ − Fs)/(F_m_’ − F_o_’) x (F_o_’/Fs). To obtain F_m_ and F_m_′, a saturating pulse light (630 nm, 8000 µmol photons m^−2^ s^−1^, 300 ms) was applied. Red actinic light (630 nm, 500 µmol photons m^−2^ s^−1^) was supplied using a chip-on-board LED array. The oxidation-reduction state of P700 in PSI was determined according to the methods of Klughammer and Schreiber [23], as follows: P_m_, total amount of photo-oxidizable P700; P_m_′, maximum amount of P700 photo-oxidized by the saturating pulse light under actinic light; P, amount of photo-oxidized P700 at a steady state under actinic light; the effective quantum yield of PSI, Y(I) = (Pm’ – P)/Pm; the quantum yield of non-photochemical energy dissipation of oxidized P700 (P700^+^), Y(ND) = P/P_m_; and the quantum yield of non-photochemical energy dissipation of photo-excited P700 (P700*), Y(NA) = (P_m_ − P_m_′)/P_m_. The summation of these quantum yields is 1 (Y(I) + Y(ND) + Y(NA) = 1).

We set the intensity of actinic light at 500 μmol photons m^−2^ s^−1^, so that we could detect Y(II) and Y(I) signals at a lower Ci. Generally, P700 is oxidized under high light and/or low CO_2_ conditions. At extremely high light (ex. >1500 μmol photons m^−2^ s^−1^), Y(II) and Y(I) are too small to allow a precise estimation of them.

For P700 in PSI to be oxidized, the reduction rate of P700^+^ must be the rate-determining step in the P700 photo-oxidation/reduction cycle. H^+^ accumulation in the lumen of thylakoid membranes, ΔpH formation, suppresses the plastoquinol (PQH_2_) oxidation of the cytochrome (Cyt) *b*_6_/*f* complex, which is called photosynthesis control, to oxidize P700 [24]. To evaluate the contribution of photorespiration to the oxidation of P700 in PSI, the electrochromic shift (ECS) signal was measured. The ECS signal reflects both the ΔpH and Δψ across the thylakoid membranes [25,26]. The ECS signal was measured simultaneously with the above gas exchange analysis using the DUAL-PAM system (Walz), equipped with a P515 analysis module [27]. The P515 analysis module monitored the formation of the ECS signal due to the carotenoid spectrum shift in response to the membrane potential produced by ΔpH and [25]. The magnitude of the ECS signal was evaluated by dark-interval relaxation kinetics (DIRK) analysis [25,26]. At the steady state of photosynthesis, actinic light (AL) illumination was transiently turned off for 400 ms. On the turning-off of AL illumination, the ECS signal rapidly decayed. The magnitude of the full decay of the ECS signal reflects the summation of both ΔpH and Δψ. The decay rate of the ECS signal after the turning-off of AL illumination reflects the activity of ATP synthase in thylakoid membranes [25,26]. The half time of the ECS decay reflects the proton conductance (gH^+^), which in turn reflects the apparent rate constant of ATP synthesis catalyzed by ATP synthase and depends on the concentrations of ADP and inorganic phosphate and the catalytic constant of ATP synthase [25,26]. 

The magnitude of the ECS signal was normalized, as follows [27]. A single turnover flash (10s) was used to illuminate the leaf under far-red light. The ECS signal was induced by the single turnover of PSII, which corresponds to the membrane potential induced by single charge separation. The average value of a single turnover (ST) flash-induced ECS signal (ECS_ST_) was 3.73 ± 0.04 × 10^−3^ ΔI/Io (*n* = 3). Then, the measured ECS signal was divided by ECS_ST_, and was used as the normalized ECS signal (ECS_N_) [25] (Equation (1)).

ECS_N_ = ECS/ECS_ST_ (1)

The contribution of both ΔpH and Δψ to the total ECS signal was separately evaluated after the turning-off of AL illumination over longer periods of darkness [26]. Under all experimental conditions in this study, the contribution of Δψ to ECS_N_ was less than 10% (Figure S1). Therefore, ECS_N_ is regarded as mainly representing ΔpH.

The H^+^ consumption flux vH^+^ (μmol H^+^ m^−2^ s^−1^) is proportional to both ECS_N_ and gH^+^. Namely (Equation (2)),

vH^+^ = m × gH^+^ × ECS_N_, (2)

where m is a coefficient that has the dimension of “mol H^+^ m^−2^”. In this study, we assumed that m was constant.

### 2.3. Ribulose 1,5-Bisphosphate (RuBP) Carboxylation Rate and RuBP Oxygenation Rate in Wheat Leaves

The RuBP carboxylation rate (vc) and RuBP oxygenation rate (vo) during photosynthesis and photorespiration in wheat leaves were measured by simultaneous chlorophyll fluorescence and CO_2_ exchange analyses [28,29]. The values for vc and vo were obtained from the following equations (Equations (3) and (4)):

vc = (1/6) × [Jf/2 + 4 × (A + Rd)],(3)

vo = (1/6) × [Jf − 4 × (A + Rd)],(4)

where Jf is the electron flux in the photosynthetic linear electron flow (LEF) and is equal to α × Y(II) × PFD [30]. The photosynthesis rate (A) and dark respiration rate (Rd) were measured as described above. The photon energy absorbed by the leaves is distributed to both PSII and PSI. The coefficient α is the distribution ratio of the photon energy to PSII in the thylakoid membrane. The value of α for wheat leaves, which was 0.42 ± 0.02 (*n* = 4) in this study, was determined following the method of Miyake and Yokota [31]. The term PFD stands for the photon flux density, which is the intensity of light illuminated on the leaves.

### 2.4. H^+^ Consumption Rate Estimated from the Stoichiometries of Photosynthesis and Photorespiration 

The H^+^ consumption rate (JgH^+^) was estimated from the ATP consumption rate (vATP) during photosynthesis and photorespiration [32]. In C3 photosynthesis, the ratio of JgH^+^ to vATP is 4.67, because ATP synthase uses 4.67 H^+^ ions for the synthesis of one molecule of ATP [33]. The ratio of vATP to the NADPH consumption rate (vNADPH) is [3 + 3.5 (vo/vc)]/[(2 + 2 (vo/vc)]. Considering JgH^+^/vNADPH = 4.67 × [3 + 3.5 (vo/vc)]/[(2 + 2 (vo/vc)] and the electrons in photosynthetic linear electron flow for the production of NADPH, JgH^+^ could be expressed as follows [21] (Equation (5)):

JgH^+^ = 9.34 × (vc + vo) × [3 + 3.5 (vo/vc)]/[2 + 2 (vo/v c)]. (5)

The values of both vc and vo were estimated as described above.

## 3. Results

### 3.1. Characteristics of PSII and PSI Parameters in Response to Changes in the Partial Pressure of CO_2_

**Figure 1:**
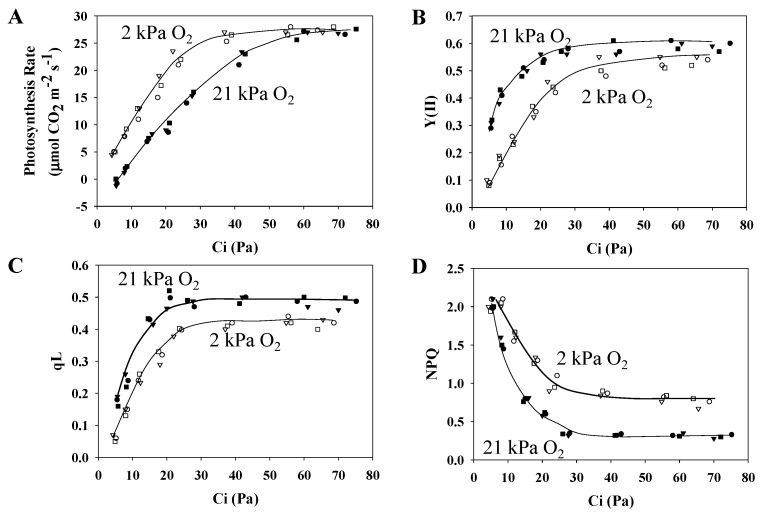
Influence of the partial pressure of O_2_ on the photosynthesis rate and chlorophyll (Chl) fluorescence parameters as a function of the partial pressure of intercellular CO_2_ (Ci) in wheat leaves. Photosynthesis rates (**A**) were measured at 21 and 2 kPa O_2_, at 500 µmol photons m^−2^ s^−1^, simultaneously with the measurement of the effective quantum yield of photosystem II (PSII) (Y(II)) (**B**), the photochemical quenching of Chl fluorescence, the Q_A_ oxidized state (qL) (**C**), and the non-photochemical quenching (NPQ) of Chl fluorescence (**D**). Data were obtained from three independent experiments using leaves attached to three wheat plants (*N* = 3: sample 1, circle; 2, square; 3, triangle). The ambient partial pressures of CO_2_ were changed from 100 to 5 through 80, 60, 40, 30, 20, and 10 Pa at 21 and 2 kPa O_2_ for the same leaves. Closed symbols, 21 kPa O_2_; open symbols, 2 kPa O_2_. Lines in the graphs were arbitrarily drawn to indicate the trends of the data.

**Figure 2:**
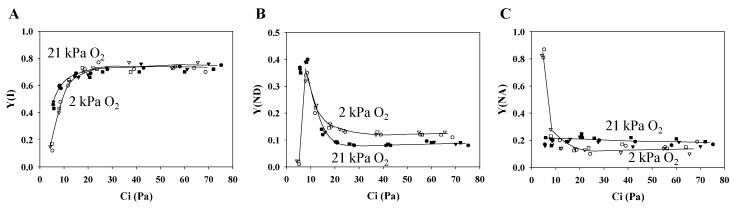
Influence of the partial pressure of O_2_ on the redox state of P700 in PSI as a function of the partial pressure of intercellular CO_2_ (Ci) in wheat leaves. The effective quantum yield of photosystem I (PSI) (Y(I)) (**A**), the oxidized state of P700 (Y(ND)) (**B**), and the excited state of P700 (Y(NA)) (**C**) were simultaneously measured with the photosynthesis rate and chlorophyll fluorescence yield measurements. Y(I) + (ND) + Y(NA) = 1. Data were obtained from three independent experiments using leaves attached to three wheat plants (*N* = 3: sample 1, circle; 2, square; 3, triangle). The ambient partial pressures of CO_2_ were changed from 100 to 5 through 80, 60, 40, 30, 20, and 10 Pa at 21 and 2 kPa O_2_ for the same leaves. Closed symbols, 21 kPa O_2_; open symbols, 2 kPa O_2_. Lines in the graphs were arbitrarily drawn to indicate the trends of the data.

To examine the effect of photorespiration on the photochemical parameters in PSII and PSI, we modulated the photorespiration rate by manipulating the partial pressure of CO_2_ (pCO_2_). Photorespiration activity is expected to increase when lowering pCO_2_ under atmospheric conditions [34,35,36], and lowering the atmospheric partial pressure of O_2_ (pO_2_) (21 kPa) to 2 kPa achieves negligible photorespiration activity [34,35,36]. We set pO_2_ to 21 kPa, pCO_2_ to 40 Pa, and the light intensity to 500 µmol photons m^−2^ s^−1^. After the photosynthesis rate reached a steady-state level, we increased pCO_2_ to 100 Pa. Next, we lowered pCO_2_ to 5 Pa from 100 Pa, and under all pCO_2_, we assessed the photosynthesis rate, along with the PSII and PSI parameters (Figure 1 and Figure 2). These assessments were conducted under two pO_2_ conditions (21 kPa, normoxic condition; 2 kPa, hypoxic conditions).

The following parameters were plotted against the leaf intercellular CO_2_ partial pressure (Ci) under the two pO_2_ conditions: the photosynthesis rate (Figure 1A), the PSII quantum yield (Y(II)) (Figure 1B), qL reflecting the Q_A_ redox state in PSII (Figure 1C), and NPQ (Figure 1D). 

The photosynthesis rate under the normoxic condition showed a CO_2_ compensation point of approximately 6 Pa pCO_2_, and the photosynthesis rate increased as Ci increased, becoming saturated at roughly 60 Pa Ci (Figure 1A). On the other hand, under the hypoxic condition, the CO_2_ compensation point decreased, and the photosynthesis rate was even greater than that under the normoxic condition. This is because, under the hypoxic condition, photorespiration was suppressed [31,36]. Y(II) also increased as Ci increased (Figure 1B). However, unlike the photosynthesis rate, Y(II) values were greater under the normoxic condition rather than under the hypoxic condition. This reflects the increased electron sink provided by photorespiration [31]. Furthermore, as with Y(II), qL showed a response to changes of both Ci and pO_2_ (Figure 1C); that is, Q_A_ was oxidized in response to Y(II) increasing, and this was caused by the increased electron sink provided by photorespiration [31]. NPQ decreased in response to the increase in Y(II) (Figure 1D). Furthermore, the increase in both Y(II) and qL facilitated by photorespiration lowered the NPQ values further under the normoxic condition than under the hypoxic condition, considering that (Equation (6)) [37]

NPQ = qL × [1 − Y(II)]/Y(II) × (Fv/Fm)/[1 − (Fv/Fm)] (6)

The quantum yields of PSI were plotted against Ci under the two pO_2_ conditions: Y(I) (Figure 2A), Y(ND) (Figure 2B), and Y(NA) (Figure 2C).

Y(I) increased as Ci increased (Figure 2A). Unlike the photosynthesis rate, the Y(I) values were approximately the same under the normoxic condition. On the other hand, under the hypoxic condition, Y(I) decreased to roughly 0.15 when Ci was lower than 5Pa, where photosynthesis and photorespiration activities were negligible. Furthermore, Y(ND), representing the oxidation level of P700, also showed a response to Ci changes (Figure 2B). Drops in Ci led to increases in Y(ND). Y(ND) under the normoxic condition was lower than under the hypoxic condition, above 15 Pa Ci. Meanwhile, under photorespiration-suppressed conditions, the suppression of photosynthesis activity in the Ci range lower than 10 Pa caused Y(ND) to fall to roughly 0.01. However, Y(ND) did not fall in the same Ci range under the normoxic condition. Y(NA) did not depend on photorespiration activity and showed no Ci response (Figure 2C), except when photorespiration was suppressed and Ci was low, where Y(NA) only increased to approximately 0.85. Below 10 Pa Ci under the hypoxic condition, photosynthesis and photorespiration, that is, almost all electron sinks, were suppressed, as shown by the extremely small Y(I) and Y(II). Because of the suppressed electron flux in both PSII and PSI, the P700 oxidation reflected in Y(ND) was suppressed and Y(NA) was enhanced.

### 3.2. Characteristics of the Electrochromic Shift Signal and H^+^ Conductance in Response to Changes in pCO_2_

**Figure 3:**
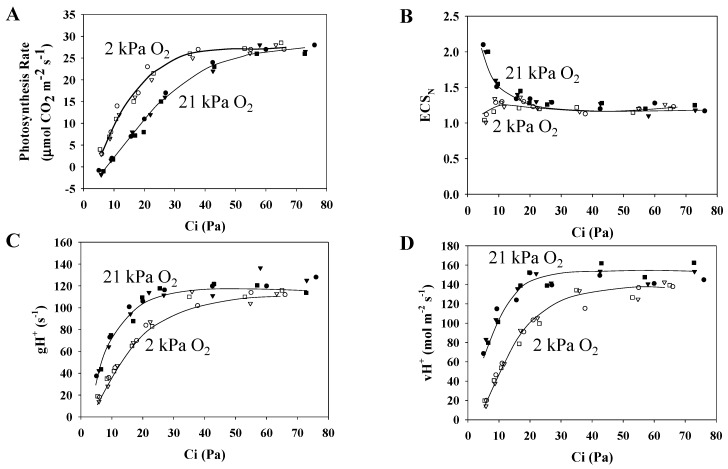
Influence of the partial pressure of O_2_ on the parameters of proton motive force reflected as an electrochromic shift (ECS) signal, H^+^ conductance (gH^+^), and the ECS decay rate (vH^+^) due to CO_2_ fixation and photorespiration as a function of the partial pressure of intercellular CO_2_ (Ci) in wheat leaves. Photosynthesis rates (**A**) were measured at 21 and 2 kPa O_2_, at 500 µmol photons m^−2^ s^−1^, simultaneously with the measurements of electrochromic shift (ECS_N_) (**B**), H^+^ conductance (gH^+^) (**C**), and the ECS decay rate (vH^+^) (**D**). Data were from three independent experiments using leaves attached to three wheat plants (*N* = 3: sample 1, circle; 2, square; 3, triangle). The ambient partial pressures of CO_2_ were changed from 100 to 5 through 80, 60, 40, 30, 20, and 10 Pa at 21 and 2 kPa O_2_ for the same leaves. Closed symbols, 21 kPa O_2_; open symbols, 2 kPa O_2_. Lines in the graphs were arbitrarily drawn to indicate the trends of the data.

To reveal the effects of photorespiration on the electrochromic shift (ECS_N_) signal and on H^+^ conductance (gH^+^), we analyzed the effects of pCO_2_ on photosynthesis in wheat leaves. The methods for analyzing the photosynthesis rate and these parameters are described in Figure 1 (Figure 3A).

The following parameters were plotted against Ci, under normoxic and hypoxic conditions: the photosynthesis rate (Figure 3A), ECS_N_ (Figure 3B), gH^+^ (Figure 3C), and the ECS_N_ decay rate (vH^+^) (Figure 3D).

ECS_N_ did not show Ci dependence in response to the photorespiration-suppressed situation of the hypoxic condition (Figure 3B). In contrast, in the photorespiration-functional situation of the normoxic condition, lowering Ci caused ECS_N_ to increase, suggesting that photorespiration contributed to ΔpH induction. Under both normoxic and hypoxic conditions, the proportion of ΔpH in the ECS_N_ was over 90%, while the proportion of ΔΨ was under 10% (Figure S1A,B). As with Y(II), gH^+^ showed Ci dependence (Figure 3C). The gH^+^ values were greater under the normoxic condition than under the hypoxic condition. Furthermore, as with both Y(II) and gH^+^, vH^+^ showed Ci dependence (Figure 3D). The value of vH^+^ was estimated by multiplying gH^+^ by ECS_N_ (see Section 2, “Materials and Methods”). These facts support that photorespiration increased vH^+^ in the thylakoid membrane, compared to the hypoxic condition.

### 3.3. Electron Flux of Photosynthetic LEF Matches the Rate of ECS Deay Driven by Photosynthesis and Photorespiration

**Figure 4:**
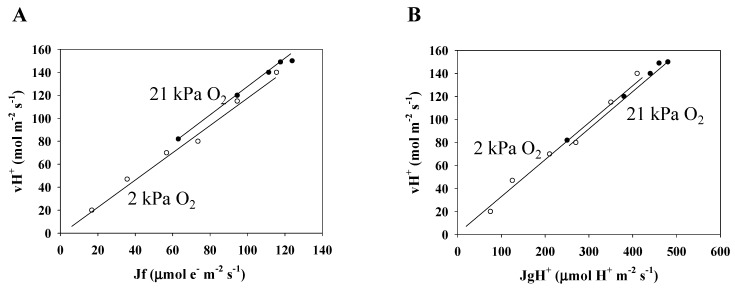
Relationships between the ECS decay rate (vH^+^) and the electron flux in photosynthetic linear electron flow (Jf), reflected as α × Y(II) × PFD, and between vH^+^ and the H^+^ consumption rate (JgH^+^). The data for vH^+^, Jf, and JgH^+^ were obtained from Figure S2 (see further details in the text). (**A**) vH^+^ was plotted against Jf. (**B**) vH^+^ was plotted against JgH^+^. Closed symbols, 21 kPa O_2_; open symbols, 2 kPa O_2_. Lines in the graphs were arbitrarily drawn to indicate the trends of the data.

We examined the relationship between the electron flux in photosynthetic linear electron flow (LEF) and the H^+^ consumption flux of both photosynthesis and photorespiration in the thylakoid membrane. In this study, Jf, reflecting LEF, and vH^+^, were not measured simultaneously. Therefore, the Jf values were plotted against A + Rd, based on Figure 1 (Figure S2A). The relationships between Jf and A + Rd are shown by the arbitrarily drawn lines, which represent the trend of the data. Furthermore, the vH^+^ values were plotted against A + Rd based on Figure 3 (Figure S2B). The relationships between vH^+^ and A + Rd are shown as the same as with Jf. In Figure S2A,B, Jf and vH^+^ were sampled at the same values of A + Rd, on the basis of the arbitrarily drawn lines. Then, vH^+^ was plotted against the Jf values (Figure 4A). Under the two pO_2_ conditions, vH^+^ showed a positive linear relationship with Jf, with an origin of zero. These results agree with those of Avenson et al. [26] Kadota et al. [38] reported that the electron flux in ferredoxin (Fd)-dependent cyclic electron flow (CEF) activity is negligible compared to the electron flux in LEF under high light intensity conditions [38]. Therefore, it is implied that in a steady state, vH^+^ is equal to the rate of H^+^ accumulation in the thylakoid lumen driven by LEF.

Next, we estimated the flux of H^+^ consumption (JgH^+^) for the regeneration of ATP that is required for driving photosynthesis and photorespiration, on the basis of the Ci dependence data for both the photosynthesis rate and Y(II) (Figure 1A,B) (see “Materials and Methods” [32]). JgH^+^ values were plotted against A + Rd (Figure S2C). In Figure S2B,C, vH^+^ and JgH^+^ were sampled at the same A + Rd values (Figure 2A,B). Then, vH^+^ was plotted against JgH^+^ (Figure 4B). Under both normoxic and hypoxic conditions, vH^+^ showed a positive linear relationship with JgH^+^, with an origin of zero. These results agree with the results of Sejima et al. [32]. These sets of results show that the vH^+^ is determined by the ATP regeneration rate in photosynthesis and photorespiration. From the fact that LEF driven by photosynthesis and photorespiration shows a clear linear relationship with vH^+^ having an origin point of zero, we can conclude that the light reaction tightly couples with the dark reaction; that is, these results also support that the activities of alternative electron flows producing ΔpH across thylakoid membranes, the water–water cycle, and/or Fd-CEF, are extremely low and/or negligible.

### 3.4. Contribution of Photorespiration to P700 Oxidation and ECS_N_ in Response to Changes in the H^+^ Consumption Rate

**Figure 5:**
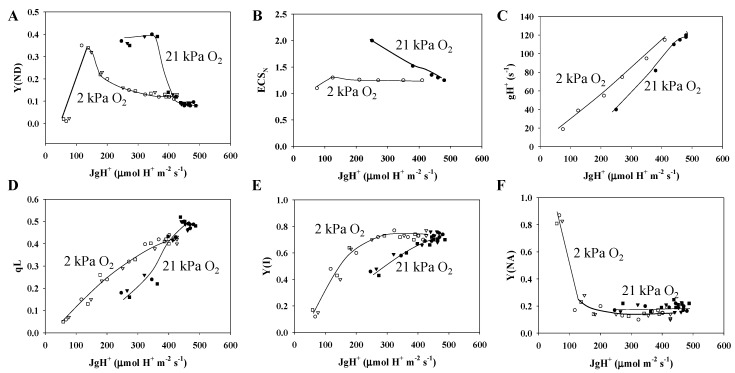
Relationships between Y(ND) and JgH^+^, ECS and JgH^+^, gH^+^ and JgH^+^, qL and JgH^+^, Y(I) and JgH^+^, and Y(NA) and JgH^+^. The data for each parameter were taken from Figure 1, Figure 2 and Figure 3, and Figure S3 (see further details in the text). (**A**) Y(ND), (**B**) ECS_N_, (**C**) gH^+^, (**D**) qL, (**E**) Y(I), and (**F**) Y(NA) were plotted against JgH^+^ at 21 and 2 kPa O_2_. Closed symbols, 21 kPa O_2_; open symbols, 2 kPa O_2_. Lines in the graphs were arbitrarily drawn to indicate the trends of the data.

The role of photorespiration in P700 oxidation was assessed (Figure 5). Under the normoxic condition, decreases in JgH^+^ from 450 to 250 μmol H^+^ m^−2^ s^−1^ induced by lowering Ci enhanced P700 oxidation, as shown by the increase in Y(ND), compared to the hypoxic condition (Figure 5A). These results indicate that photorespiration contributes to the oxidation of P700 in PSI. We tried to clarify the molecular mechanism required to oxidize P700 by photorespiration, for which the reduction of P700^+^ should be suppressed in the P700 photo-oxidation reduction cycle. The PQH_2_ oxidation activity exhibited by the Cyt *b*_6_/*f* complex is suppressed by the acidification of the luminal space of thylakoid membranes and RISE, which contribute to the suppression of the reduction of P700^+^ in PSI. A decrease in JgH^+^ induces a reduction of PQ-pool, as shown by the decrease in qL under the normoxic condition, the extent of which was larger than that under the hypoxic condition (Figure 5D). These results correspond to those of Shaku et al. [8] and Shimakawa, Shaku et al. [10]. One of the molecular mechanisms for the oxidation of P700 is RISE [10]. Compared to the hypoxic condition, qL decreased much more under the normoxic condition (Figure 5D). The range of the smaller qL under the normoxic condition compared to the hypoxic condition corresponds to the range of the larger Y(ND). On the other hand, the acidification of the luminal space of thylakoid membranes suppresses PQH_2_ oxidation activity of the Cyt *b*_6_/*f* complex [24]. The ΔpH was evaluated as the ECS_N_ increased in response to the decrease in JgH^+^ under the normoxic condition, but did not change under the hypoxic condition (Figure 5B). That is, photorespiration stimulated the formation of ΔpH across thylakoid membranes to suppress the PQH_2_ oxidation activity of the Cyt *b*_6_/*f* complex [24] and enhance the oxidation of P700. The reason why the ΔpH increased under the normoxic condition could be because the values of gH^+^ were lower than those under the hypoxic condition (Figure 5C). These facts suggest that the regulatory mechanism lowers the H^+^ conductance of thylakoid membranes by photorespiration.

Under the normoxic condition, decreases in JgH^+^ from 450 to 250 μmol H^+^ m^−2^ s^−1^ lowered the electron flux in PSI, as shown in the decrease in Y(I), compared to the hypoxic condition (Figure 5E). These results indicate that photorespiration suppresses the electron flux in PSI by enhancing the oxidation of P700, because Y(NA) did not change (Figure 5F); that is, the suppression of the photosynthetic linear electron flow from the Cyt *b*_6_/*f* complex to PSI gets preference over the activity of PSI under the normoxic condition. The oxidation of P700 lowers the chance of O_2_ being reduced to O_2_^−^ at the acceptor side of PSI by decreasing Y(I) and keeping Y(NA) at a lower value. Under the hypoxic condition, Y(I) further decreased with the increase in Y(NA) below 250 μmol H^+^ m^−2^ s^−1^ (Figure 5E,B). The increase in Y(NA), reflecting the accumulation of electrons at the acceptor side of PSI, is not dangerous for PSI, because the probability of producing ROS is too small under the hypoxic condition [3].

**Figure 6:**
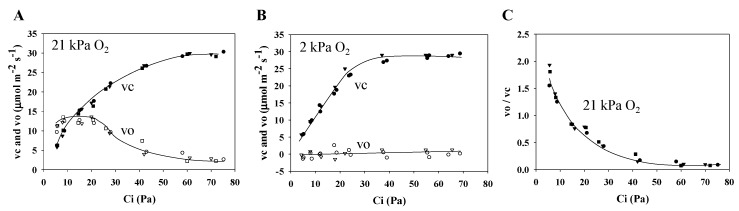
Influence of the intercellular partial pressure of CO_2_ (Ci) on both the ribulose 1,5-bisphosphate (RuBP) carboxylase reaction rate (vc) and the RuBP oxygenase reaction rate (vo) in wheat leaves. Photosynthesis rates were measured at 21 and 2 kPa O_2_, at 500 µmol photons m^−2^ s^−1^, simultaneously with the measurement of chlorophyll fluorescence. Both vc and vo were estimated from the photosynthesis rates and the values of Y(II) [21]. Data were obtained from Figure 1 (sample 1, circle; 2, square; 3, triangle). (**A**) Both vc and vo were plotted against Ci at 21 kPa O_2_. Closed symbols, vc; open symbols, vo. (**B**) Both vc and vo were plotted against Ci at 2 kPa O_2_. Closed symbols, vc; open symbols, vo. (**C**) The values of vo/vc at 21 kPa O_2_ were plotted against Ci. Lines in the graphs were arbitrarily drawn to indicate the trends of the data.

The results detailed above show that photorespiration contributes to P700 oxidation. The Ci dependencies of Rubisco’s vc and vo were plotted under the normoxic and hypoxic conditions (Figure 6A,B). Furthermore, vo/vc was plotted against Ci (Figure 6C). These results show that vo increases owing to the decrease in Ci, and that photorespiration activity increases under the normoxic condition. Interestingly, when Ci < 20 Pa, Ci decreases do not increase the photorespiration activity. These results agree with the results of Miyake and Yokota [31]. The causes of the suppression of the increase in photorespiration activity with the Ci drop will be discussed in the Discussion section. In contrast, the photorespiration activity was negligible under the hypoxic condition (Figure 6B).

## 4. Discussion

In the present research, we tried to elucidate the physiological function of photorespiration in P700 oxidation in PSI. Generally, P700 is oxidized by the limitation of electron flow to the oxidized form of P700, P700^+^ [1], and the oxidation activity of PQH_2_ of the cytochrome *b*_6_/*f* complex is down-regulated by the acidification of the luminal space (photosynthetic control) and RISE. We compared the relationship between P700 oxidation and photorespiration in terms of both photosynthetic control and RISE. We clarified that photorespiration decreased the H^+^ conductance and gH^+^, induced ΔpH formation, and simultaneously enhanced the reduction of PQ. These facts show that the rate of ATP consumption in photorespiration would be lower than that in photosynthesis; that is, a metabolic transition from only photosynthesis to both photosynthesis and photorespiration would cause a decrease in the efficiency of the regeneration of ATP due to photorespiration. The enhanced ΔpH formation induced the reduction of PQ to further suppress the PQH_2_ oxidation activity of the Cyt *b*_6_/*f* complex. Photorespiration might induce RISE by ΔpH formation.

We next considered the molecular mechanism for ΔpH formation across the thylakoid membranes to understand the P700 oxidation mechanism. In C3 angiosperms, the electron flux in photosynthetic LEF showed a positive linear relationship (with an origin of zero) with electron consumption rates (Jg) in both photosynthesis and photorespiration [30,39]. These results show that photosynthetic LEF drives both photosynthesis and photorespiration activity. Furthermore, we recently found that the LEF rate and Fd oxidation rate have a similar relationship to that between the LEF rate and Jg [38]. These results show that ferredoxin (Fd)-dependent CEF [40,41,42,43,44,45,46] is negligibly small; that is, photosynthetic LEF is responsible for the majority of ΔpH formation [38]. The induction mechanism of ΔpH formation across the thylakoid membranes is shown in the following manner: ΔpH formation is observed as an ECS signal increase [26,47]. Then, the ECS generation and decay rate [d(ECS)/dt] are determined by the difference between the ECS generation rate dependent on the LEF flux (Jf = α × Y(II) × PFD, see the detail in “Materials and Methods”) and the ECS decay rate (vH^+^) of the ATP regeneration reaction required for photosynthesis and photorespiration (Equations (7) and (8)),:

d(m × ECS_N_)/dt = k × Jf − vH^+^, (7)

= k × Jf − m × gH^+^ × ECS_N_. (8)

The coefficient k reflects H^+^ accumulation in the lumens, which is driven by LEF, and depends on H_2_O oxidation in PSII and on Q-cycle rotation in the Cyt *b*_6_/*f* complex. Furthermore, vH^+^ is expressed as m × gH^+^ × (ECS_N_). The gH^+^, H^+^ conductance is a rate constant that reflects the apparent rate constant of ECS decay. The vH^+^ reflects the ΔpH dissipation rate, and vH^+^ can be replaced with JgH^+^ as follows (Equation (9)):

d(m × ECS_N_)/dt = k × Jf − JgH^+^. (9)

The validity of vH^+^ = JgH^+^ is provided by the fact that the relationship between the two in a steady state is shown to be positive and linear, with an origin of zero (Figure 4B). This confirms that vH^+^ is equal to the H^+^ usage rate for the ATP regeneration required for photosynthesis and photorespiration. These results agree with the results of [32].

We could confirm that, in a steady state where [d(m × ECS_N_)/dt = 0], vH^+^ shows a positive linear relationship with the LEF rate, with an origin of zero (Figure 4A). These results agree with the results of [26]. Therefore, the fact that vH^+^ reflects JgH^+^ shows that the ATP consumed in photosynthesis and photorespiration can only be supplied by LEF; that is to say, the following relationship is proposed (Equation (10)):

k × Jf = m × gH^+^ × ECS_N_ = JgH^+^. (10)

Equation (4) shows that LEF activity links photosynthesis and photorespiration activity through ΔpH formation and dissipation. From these results (Equations (11) and (12)),

ECS_N_ = (k × Jf)/(m × gH^+^), (11)

= JgH^+^/(m × gH^+^). (12)

Based on this model, we will discuss the molecular mechanism of P700 oxidation.

The primary causes of P700 oxidation under the hypoxic condition, in which only photosynthesis functions, can be explained as follows. Decreases in JgH^+^ gradually oxidized P700 (Figure 5A). However, the ECS_N_ values remained the same (Figure 5B). Meanwhile, decreases in JgH^+^ lowered gH^+^ (Figure 5C). The ratio of the JgH^+^ decrease was equal to the ratio of the gH^+^ decrease. This is the reason that ECS_N_ remained constant (equations (5) and (6)). We found that qL decreased along with decreases in JgH^+^ (Figure 5D). This shows that the PQ pool is reduced along with the lowering of Jf [48]. This may be the reason why RISE is induced [1,8,10,49]. RISE caused by PQ reduction induces P700 oxidation by lowering the activity of PQH_2_ oxidation of the Cyt *b*_6_/*f* complex.

Next, we attempted to elucidate how photorespiration contributes to the oxidation of P700 in PSI. In the photorespiratory situation under the normoxic condition, the decrease in JgH^+^ from 400 to 200 μmol H^+^ m^−2^ s^−1^ enhanced the increase in Y(ND) compared to the non-photorespiratory situation under the hypoxic condition (Figure 5A). ECS_N_ also increased, which was due to the enhanced decrease in gH^+^, compared to the decrease in JgH^+^ (Figure 5C; Equation (6)). Furthermore, qL also decreased under the normoxic condition compared to the hypoxic condition (Figure 5D); that is, photorespiration oxidized P700 by photosynthetic control through ΔpH formation and RISE through PQ reduction.

In this study, we discovered important facts about photorespiration. Under the normoxic condition, the values of gH^+^ were lower compared to under the hypoxic condition, in the range of JgH^+^ from 250 to 400 μmol H^+^ m^−2^ s^−1^ (Figure 5C). This fact shows that the activity of ATP synthase might decrease under the normoxic condition. The detailed mechanism for this remains to be clarified.

When photorespiration functions, both gH^+^ and qL decrease, both of which induce photosynthetic control and RISE (Figure 5C,D). This contributes to the oxidation of P700 in PSI, as described above. On the other hand, we found suppressed rates of the ribulose-1,5-bisphosphate (RuBP) carboxylase reaction (vc) and RuBP oxygenase reaction (vo), catalyzed by RuBP carboxylase/oxygenase (Rubisco) (Figure 6). Following Rubisco kinetics, a decrease in Ci should cause an increase in vo [34,35,36]. The data in Figure 6 correspond to the results of Miyake and Yokota [31]. RISE has the potential to lower LEF activity while simultaneously contributing to P700 oxidation [8]. These facts show that the photosynthetic electron transport reaction, a light reaction, regulates both photosynthesis and photorespiration with the oxidation of P700 in PSI. 

## Supplementary Materials

The following are available online at https://www.mdpi.com/2223-7747/9/3/319/s1, Figure S1: Both ΔpH and ΔΨ which contribute to proton motive force (pmf), reflected as the total electrochromic shift (ECS) signal, were separately determined with ECS in Figure 3 following the method of Cruz et al. (2001), Figure S2: Relationships of Y(II), vH^+^, JgH^+^, ECS, and gH^+^ with (A + Rd), Figure S3: Dependence of Jf and JgH^+^ on Ci, and the relationship between JgH^+^ and Jf.
