# Supplementary material for: Photorespiration Enhances Acidification of the Thylakoid Lumen, Reduces the Plastoquinone Pool, and Contributes to the Oxidation of P700 at a Lower Partial Pressure of CO2 in Wheat Leaves
Source: Plants (Basel). 2020 Mar 3;9(3):319. doi: 10.3390/plants9030319 (PMC7154841; doi:10.3390/plants9030319)
Supplement: Supplementary file 1 [file plants-09-00319-s001.pdf]

**Supplementary Materials**

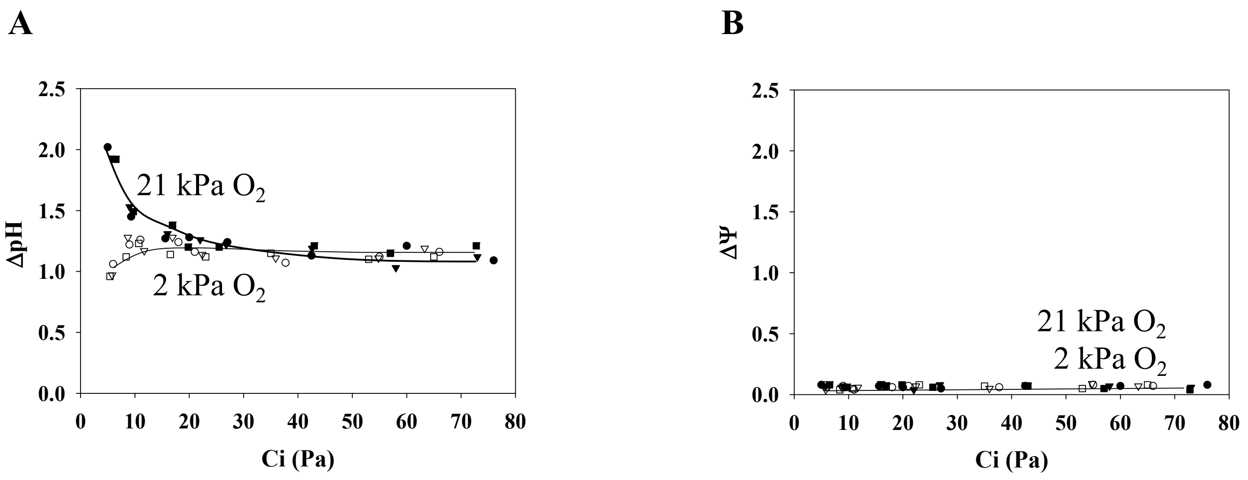


**Figure S1.** Both ΔpH and ΔΨ which contribute to proton motive force (pmf), reflected as the total electrochromic shift (ECS) signal, were separately determined with ECS in Figure 3 following the method of Cruz et al. (2001). Please see the details in the text. (**A**), ΔpH; (**B**) ΔΨ. Closed circle, 21 kPa $O_2$; Open circle, 2 kPa $O_2$. Data were from three independent experiments using leaves attached to three wheat plants (N = 3: sample 1, circle; 2, square; 3, triangle). The ambient partial pressures of $CO_2$ were changed from 100 to 5 through 80, 60, 40, 30, 20, and 10 Pa at 21 and 2 kPa $O_2$, for the same leaves. Lines in the graphs were arbitrarily drawn to indicate the trends of the data.

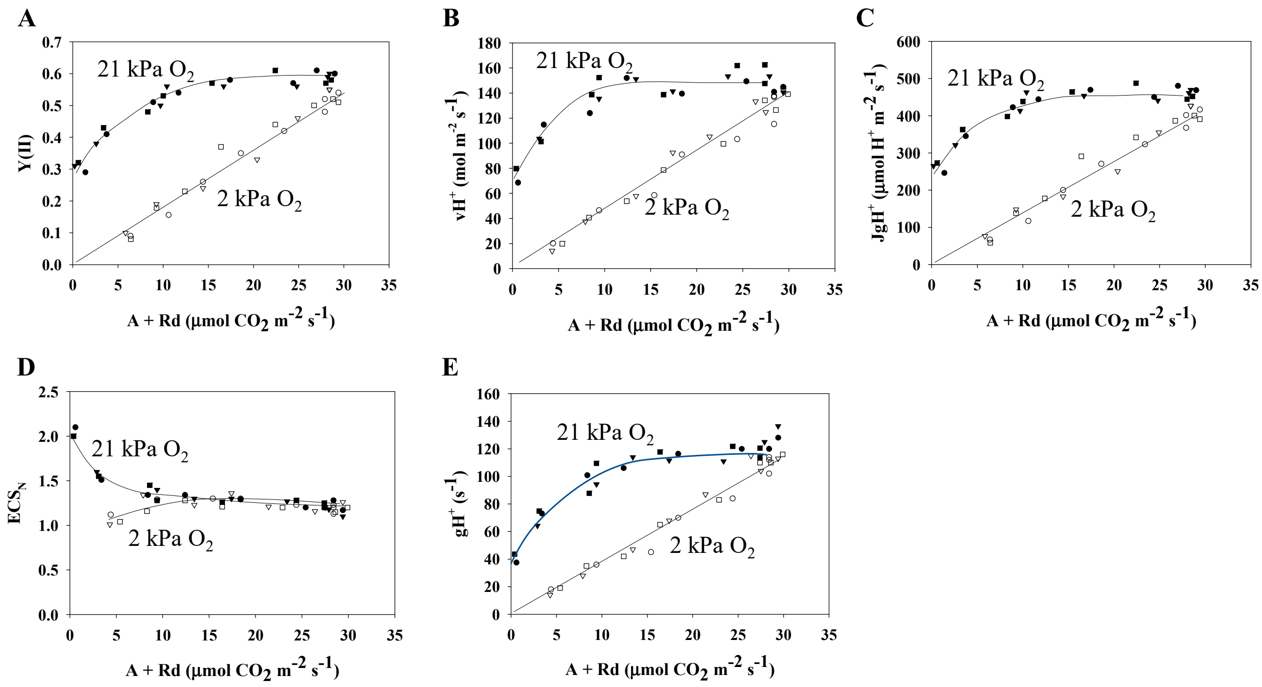


**Figure S2.** Relationships of Y(II), $vH^+$, $JgH^+$, ECS, and $gH^+$ with (A + Rd). The data for Y(II) and (A + Rd) were from Figures 1 and 2, and $JgH^+$ was calculated from the data in Figure 1 by the method, described in the "Materials and Methods". The data for ECS, $vH^+$, $gH^+$, and (A + Rd) were from Figure 3. (**A**) Y(II) was plotted against (A + Rd). (**B**) $vH^+$ was plotted against (A + Rd). (**C**) $JgH^+$ was plotted against (A + Rd). (**D**) ECS was plotted against (A + Rd). (**E**) $gH^+$ was plotted against (A + Rd). Data were from three independent experiments using leaves attached to three wheat plants (N = 3: sample 1, circle; 2, square; 3, triangle). The ambient partial pressures of $CO_2$ were changed from 100 to 5 through 80, 60, 40, 30, 20, and 10 Pa at the two $pO_2$ conditions (closed symbols, 21 kPa; open symbols, 2 kPa), for the same leaves. Lines in the graphs were arbitrarily drawn to indicate the trends of the data.

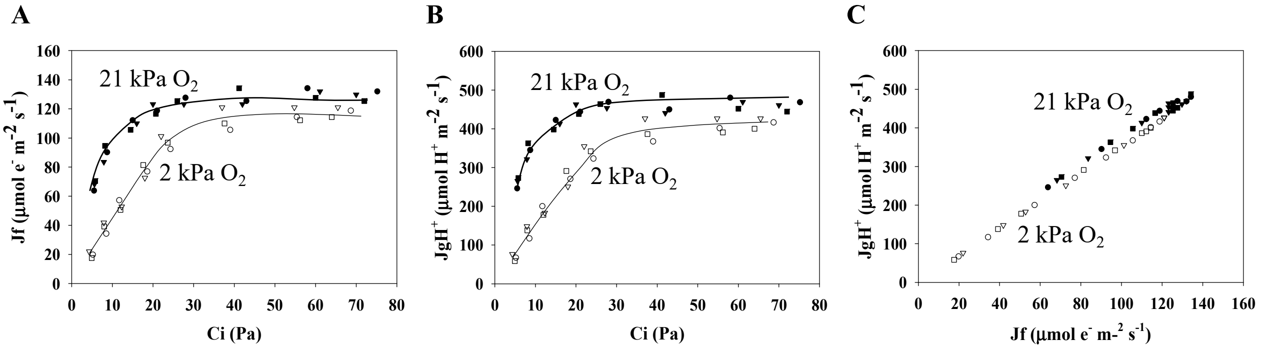


**Figure S3.** Dependence of Jf and $JgH^+$ on Ci, and the relationship between $JgH^+$ and Jf. The electron flux in photosynthetic linear electron flow (Jf), reflected as the electron flux in PSII [Y(II)], was calculated as $\alpha$ × Y(II) × PFD (please see the detail in "Materials and Methods"). The data for Y(II) and $JgH^+$ were from Figure 1. (**A**) Jf was plotted against Ci. (**B**) $JgH^+$ was plotted against Ci. (**C**) $JgH^+$ was plotted against Jf, both of which were from Supplementary Figures 3A and 3B. Data were from three independent experiments using leaves attached to three wheat plants (N = 3: sample 1, circle; 2, square; 3, triangle). The ambient partial pressures of $CO_2$ were changed from 100 to 5 through 80, 60, 40, 30, 20, and 10 Pa at 21 (closed symbols) and 2 kPa $O_2$ (open symbols), for the same leaves. Lines in the graphs were arbitrarily drawn to indicate the trends of the data.
